# Extracellular Matrix Alterations in Metastatic Processes

**DOI:** 10.3390/ijms20194947

**Published:** 2019-10-07

**Authors:** Mayra Paolillo, Sergio Schinelli

**Affiliations:** Department of Drug Sciences, University of Pavia, 27100 Pavia, Italy; sergio.schinelli@unipv.it

**Keywords:** ECM components, collagen, fibronectin, cancer associated fibroblasts, tumor associated macrophages, pre-metastatic niche

## Abstract

The extracellular matrix (ECM) is a complex network of extracellular-secreted macromolecules, such as collagen, enzymes and glycoproteins, whose main functions deal with structural scaffolding and biochemical support of cells and tissues. ECM homeostasis is essential for organ development and functioning under physiological conditions, while its sustained modification or dysregulation can result in pathological conditions. During cancer progression, epithelial tumor cells may undergo epithelial-to-mesenchymal transition (EMT), a morphological and functional remodeling, that deeply alters tumor cell features, leading to loss of epithelial markers (i.e., E-cadherin), changes in cell polarity and intercellular junctions and increase of mesenchymal markers (i.e., N-cadherin, fibronectin and vimentin). This process enhances cancer cell detachment from the original tumor mass and invasiveness, which are necessary for metastasis onset, thus allowing cancer cells to enter the bloodstream or lymphatic flow and colonize distant sites. The mechanisms that lead to development of metastases in specific sites are still largely obscure but modifications occurring in target tissue ECM are being intensively studied. Matrix metalloproteases and several adhesion receptors, among which integrins play a key role, are involved in metastasis-linked ECM modifications. In addition, cells involved in the metastatic niche formation, like cancer associated fibroblasts (CAF) and tumor associated macrophages (TAM), have been found to play crucial roles in ECM alterations aimed at promoting cancer cells adhesion and growth. In this review we focus on molecular mechanisms of ECM modifications occurring during cancer progression and metastatic dissemination to distant sites, with special attention to lung, liver and bone. Moreover, the functional role of cells forming the tumor niche will also be reviewed in light of the most recent findings.

## 1. Extracellular Matrix Structure and Function

The extracellular matrix (ECM), for years considered as a mere support structure for tissue architecture, is actually a dynamic compartment that modulates and regulates cell functions such as adhesion, migration, proliferation and differentiation [1]. It is an intricate network composed of approximately 300 proteins that vary in a cell/tissue-specific manner. Remodeling, production and degradation of its components can deeply influence cell and organ functions. In fact, on the basis of the relative amounts and organization of the different ECM constituents, ECM displays specific features for each tissue to support and modulate cell functions. The structural and functional differences in ECM composition determine physical and biomechanical properties of the ECM such as stiffness and mesh size, thus allowing cells to respond to mechanical stimuli in the surrounding environment [2].

The ECM is mainly composed of proteoglycans (PGs) and fibrous proteins [3,4]. PGs form a hydrated gel that fills the extracellular interstitial space and play a fundamental role in regulating tissue buffering and hydration, force-resistance tissue properties, also modulating cell binding to ECM. The most represented and studied proteins in the ECM architecture are collagens, elastins, fibronectins and laminins.

Collagen is composed of three peptidic α chains, forming a triple helical structure. In vertebrates, 46 different collagen chains can assemble to form 28 different collagen types [5]. The triple-stranded α helix can, in turn, assemble to form supramolecular structures, such as fibrils and networks.

Elastin, another abundantly expressed protein in ECM, is secreted as a precursor protein, tropoelastin, that assembles in fibers with a great number of crosslinks due to the presence of lysine residues. In tissues subjected to mechanical stretch, to absorb the mechanical stress, elastin fibers are tightly associated to collagen fibrils [6].

Fibronectin (FN) is secreted as a dimer formed by two chains joined by two C-terminal disulfide bonds, with binding sites to other ECM components such as FN dimers and collagen, and also to cell-surface integrin receptors [7]. FN, in fact, contains the RGD (Arg-Gly-Asp) motif that binds the RGD-binding integrin family [8,9].

Non-activated tissue fibroblasts synthesize and release ECM components such as type I and III collagens, elastin, fibronectin, tenascin and a variety of PGs that combine to form a network of fibers embedded in a hydrogel of PGs. Consequently, this network of protein fibers and PGs regulates the homeostasis of cells, tissues and organs and allows the ECM to resist a wide range of mechanical stresses.

It is therefore not surprising that alterations in a specific ECM component or in the interactions with adhesion receptors can have a remarkable impact on the biochemical and physical properties of the ECM and eventually lead to dysregulation of cell adhesion and function.

## 2. The Metastatic Process

Tumors can be defined as a loss of tissue organization and aberrant behavior of some cellular types that, growing independently from the surrounding tissue [10], induce changes in ECM similar to those found in wounds that fail to heal [11,12]. While early tumor detection and diagnosis are irreplaceable tools in cancer management, currently the most critical issue in tumor therapy is represented by limiting the dissemination of malignant tumors, due to their ability to spread from original solid mass and then colonize distant sites, giving rise to the metastatic process.

Briefly, the development of metastasis could be described as a complex five-steps process: (1) invasion, (2) intravasation, (3) cell survival in the circulation, (4) extravasation and (5) formation of premetastatic niche and secondary tumors growth [13]. Key players in this process are circulating tumor cells (CTC) that, upon detaching from the original tumor mass, achieve specific features necessary to survive in the bloodstream and to give rise to metastatic colonization. Several studies have demonstrated that the most important feature required for the dissemination and survival of CTCs is represented by a de-differentiation process resulting in gaining of a specific and tumor type-dependent stemness pattern. Tumor cell plasticity during all the above steps is supported by two main mechanisms: epithelial to mesenchymal transition (EMT) and its reverse counterpart mesenchymal to epithelial transition (MET).

The EMT process has been recognized as a biological cell reprogramming characterized by loss of cell adhesion, inhibition of E-cadherin expression and increased cell mobility [14]. EMT is essential during development and is regulated by several transcription factors, many of which are actually considered EMT markers: zinc finger protein snail 1 (SNAI1, SNAI2), zinc finger E-box-binding homeobox 1 (ZEB1, ZEB2), TWIST, forkhead box protein 1 (FOXC1), FOXC2, homeobox protein goosecoid (GSC), N-cadherin, vimentin and fibronectin-1 [14,15]. However, EMT and MET processes, though extensively studied, are still far from being completely understood; the prevailing hypothesis claims that EMT operates in the first steps to form CTCs that, upon survival in the circulation, after reaching the appropriate organ site, undergo MET thus gaining appropriate features required to prepare the soil as premetastatic niche and promote tumor progression [16].

Although the entire metastatic cycle may represent a potential therapeutic target, the main efforts of researchers are aimed at interfering with the molecular pathways involved in the formation of the pre-metastatic niche. In this step of the process, interactions between tumor cells and the novel microenvironment are largely influenced by the ECM composition and ECM components play key roles in the niche formation; for these reasons, studies on the molecular mechanisms supporting these interactions and ECM alterations are necessary to exploit new avenues in the therapeutic approach to cancer metastasis.

## 3. Premetastatic Niche and Tumor Microenvironment

**Figure 1:**
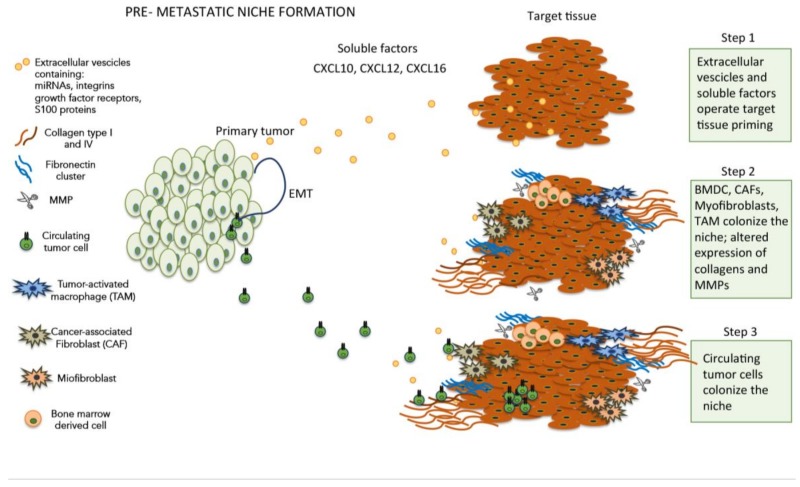
Cellular and soluble components involved in extracellular matrix (ECM) modifications during pre-metastatic niche formation.

In order to prepare the appropriate soil for the colonization of distant sites, metastatic tumor cells set in motion a complex mechanism aimed at promoting the formation of a suitable microenvironment, thereby creating the conditions for attachment, survival and growth of CTCs (Figure 1). This peculiar microenvironment, termed pre-metastatic niche [17], represents an abnormal, tumor growth-favoring microenvironment devoid of cancer cells. Several soluble factors and cells have been identified in the pre-metastatic niche formation, such as growth factors secreted by tumor cells, bone marrow-derived cells (BMDCs), extracellular vesicles (EVs), suppressive immune cells and host tissue stromal cells [18]. In more detail, the primary tumor cells were found to secrete pro-inflammatory factors such as vascular endothelial growth factor A (VEGF-A), transforming growth factor β (TGFβ) and tumor necrosis factor α (TNFα) that, in turn, induce the expression of chemoattractants such as S100A8 and S100A9 (a calcium-binding protein family) [19,20,21]. The increased expression of S100A8/S100A9 promotes cell proliferation and inhibits cell differentiation and apoptosis, thus contributing to the onset of a metastasis favorable environment. However, at the present time, the exact mechanism by which the overexpression of these chemoattractants elicits these cellular effects is still unknown. In other studies, the S100 protein and the recruitment of bone-marrow-derived myeloid cells in response to tumor-secreted factors [22,23], cooperate with fibronectin upregulation in lung resident cells to shape the pre-metastatic niche. The intriguing hypothesis assuming that soluble factors released by primary tumors could establish a favorable microenvironment promoting the growth of disseminated tumor cells was further strengthened by a seminal study [24]. In this study, the authors demonstrated that tumor-secreted exosomes, expressing different integrins subtypes on their membranes, are selectively taken up by distant non-tumor cells in a tissue-specific way, thereby contributing to prepare the soil for metastatic niche formation. Although the S100 protein was a major component of the tumor-secreted exosomes, its precise role in this mechanism remains to be disclosed.

## 4. The Tumor Microenvironment and ECM Modifications

The tumor microenvironment is formed by a wide variety of cells deputated to specific tasks in a strictly spatial defined context, such as fibroblasts, endothelial cells, pericytes, macrophages and other immune cells [25]. Nearly all the cellular components of the tumor microenvironment, especially stromal cells, contribute to metastatic cell invasion by different mechanisms, which converge on ECM modifications.

### 4.1. Fibroblasts

Tumors are generally stiffer than the normal tissue, with surrounding ECM alterations mainly induced by fibroblasts; fibroblasts are responsible for deposition and remodeling of ECM proteins [26] and facilitate tumor cell invasion through force- and protease-mediated ECM remodeling [27,28]. Moreover, growth factors and chemokines produced by cancer and endothelial cells promote stromal fibroblast activation, infiltration of T lymphocytes, macrophage activation and fibroblasts differentiation into myofibroblasts and cancer associated fibroblasts (CAFs) [28]. Myofibroblasts and CAFs, found in the stroma of carcinomas and especially in the tumor invasive edge, secrete ECM proteins, cytokines, growth factors, chemokines, hormones and inflammation proteins, thus promoting cancer cell proliferation and CSCs de-differentiation and migration [29,30]. The main contribution of CAFs and myofibroblasts to tumor microenvironment is therefore the synthesis and release of significant amounts of ECM proteins; newly deposited collagen and elastin fibers, in fact, are reoriented and remodeled to generate larger, more-rigid fibrils that contribute to the tumor-surrounding ECM special features [31,32].

### 4.2. Tumor Associate Macrophages (TAM)

Activated TAMs contribute to tumor progression by producing pro-inflammatory cytokines, such as IL-6, thus inducing a wide variety of genes involved in tumor progression and apoptosis suppression [33]. Other roles proposed for TAMs in cancer progression include VEGF, CXCL13, CCL16 and CCL18 secretion, underlining the relevance of these cells in the regulation of tumor microenvironment [34].

However, TAMs were also found to directly contribute to tumor niche formation and tumor ECM shaping by producing proteolytic enzymes (MMP-2 and MMP-9) and matrix-associated proteins. In a mouse colorectal cancer model, TAMs were found to play a role in the deposition, cross-linking and linearization of ECM collagen fibers, thus showing that the cancer ECM collagen deposition is not solely deputed to CAFs [35].

### 4.3. Pericytes (PC)

PCs reside within the wall of post-capillary venules and microvascular structures and, like other stromal components, they synthesize and release several ECM proteins. PCs play several distinct roles in the survival of cell belonging to the tumor niche: they spare endothelial cells (EC) from apoptotic death elicited by action of exogenous interleukin-12 and block the proapoptotic effect induced by tumor cell-secreted VEGF [36].

A second potential role of PCs relates to cancer microvessels formation; newly formed EC sprouts from small tumor vessels initially lack PCs and, subsequently, pericyte recruitment around these new vessels reduces EC proliferation and sprouting, leading to the formation of larger microvessels [37,38]. In a recent study, uveal melanoma and Ewing sarcoma cell lines were found to mimic the behavior of normal blood vessels by producing PDGF and recruiting PCs to induce the formation of vascular-like networks [39]. In a collagen VI-null mouse model, the basement membrane deficits deriving from the lack of collagen VI, deeply impair tumor vessel development and function thus reducing tumor growth [40].

### 4.4. Matrix Metallo Proteinases (MMPs)

As discussed above, tumor cells, CAFs, myofibroblasts, macrophages and other stromal components promote cancer cell invasion by secreting MMPs that, in turn, lead to ECM degradation, a necessary step to allow cancer cell invasion (Table 1) [41,42]. The MMP-mediated ECM modifications affect tumor evolution by facilitating the growth of solid tumor mass with different mechanisms that involve an increase of matrix stiffening and of interstitial fluid pressure. MMPs are zinc-dependent ECM remodeling endopeptidases deeply implicated in almost all steps of metastasis and a high MMP expression in tumors correlates with poor prognosis and increased risk of recurrence [1]. Different mechanisms have been shown by which MMPs alter ECM composition and promote cancer progression: for example, denaturation of fibrillar collagen stimulates melanoma cell proliferation through down-regulation of p27kip1 [43]. MMPs also contribute to the release ECM-embedded growth factors [44] and, among factors released by MMPs, VEGF plays a key role in vascular homeostasis by stimulating the proliferation of surrounding cells, promoting the formation of new blood vessels and enhancing vascular permeability [45].

The ADAM (a disintegrin and metalloproteinases) family members, including ADAM8, ADAM9, ADAM10, ADAM12 and ADAM15, exert their action by degrading the ECM proteins collagen IV and FN, thus contributing to niche formation and cancer progression [1].

ADAM8, besides cleaving important ECM components of the tumor stroma such as collagen I, fibronectin and periostin [46], can cluster with β1 integrin and could direct tumor cell invasion through localized proteolytic ECM degradation in protrusions of cancer cells [47,48]. Moreover, ADAM17 expression has been proposed as a prognostic marker for gastric cancer because this enzyme promotes metastasis and progression via activation of the Notch or Wnt signaling pathways [49].

Another MMP sub-type that can modify ECM components is represented by meprins; these enzymes cleave ECM proteins such as collagen IV and FN and can also indirectly regulate ECM remodeling by activating other metalloproteinases [1]. For example, ADAM10 is cleaved by meprin-β, and both meprin-α and meprin-β promote the cleavage of pro-MMP9 by MMP3, thus accelerating the activation of MMP9 [50]. Meprin α is expressed in several different tumors and can be secreted leading to an accumulation of meprin α in the tumor stroma [51]. For these reasons, meprin α is thought to alter ECM components structure, thereby affecting proliferation and migration of tumor cells into the surrounding tissue. However, since meprin α expression differs between different carcinomas [51], the role of meprins in cancer progression and metastasis seems to be more complex than a simple ECM-degrading function, thus requiring further studies to clarify the issue.

Another family of MMPs recently found to be implicated in cancer progression is represented by bone morphogenetic protein (BPM)/tolloid-like proteinases (BTPs) [52]. These enzymes are involved in the maturation of procollagens I–III, of fibrillar collagens V and XI and cleave procollagens V and XI. A member of the BTP family, BMP1, has been recently shown to be associated with tumor metastasis and found to be secreted by CAFs in colorectal cancer [1]; downregulation of BMP1 by microRNA miR-194 reduces lung metastasis, probably by decreasing TGF-β activation [53], while BMP1 expression predicts poor progression in clear cell renal cell carcinoma patients [54].

The amount of experimental and clinical evidence associating MMPs with tumor progression prompted efforts in synthesizing compounds targeting MMPs to be tested in clinical trials as MMP inhibitors (MMPIs) for various cancer types [55]. In spite of the large efforts made, MMPIs uniformly failed to demonstrate a survival benefit and, more importantly, severe side effects were reported. The reasons for this failure are still debated and probably administration of MMPIs in the early cancer stages rather than in stage IV could open new perspectives. However, at the moment no tangible prospect of clinical use for MMPIs are in sight.

## 5. Other Mechanisms Involved in ECM Alteration in Cancer Progression

A number of studies highlights mechanisms involved in cancer progression that require or are the consequence of ECM alterations. Mutated KRAS, for example, is known to promote squamous cell carcinoma proliferation through the MEK pathway; nevertheless, malignant transformation has been found to depend upon RAS/RHO-associated protein kinase (ROCK)-induced matrix remodeling and stiffening [56,57].

ECM stiffness directly modulates ErbB receptor dependent PI3K activation and regulates malignant mammary epithelial cells (MEC) invasion [58]. Phosphatase and tensin homolog (PTEN) is a protein-phosphatase frequently mutated or deleted in human cancers that exerts its action on cellular functionality by dephosphorylating PI3, necessary for PI3K activation, and thus negatively regulating its activity [59]. The missing link between ECM, PI3K and PTEN in MEC was identified in a mechanically-regulated miRNA, miR-18a, which targets PTEN and reduces its expression, thereby promoting PI3K-dependent malignancy [58].

Another cancer type in which an important contribution of stromal ECM is involved in tumor progression is pancreatic ductal adenocarcinoma (PDAC). Notably, in PDAC cells a loss of TGF-β signaling, in part mediated by elevated integrin β-1 mechanosignaling, elicits a positive loop in which the STAT3-dependent-signaling pathway promotes tumor progression by increasing matricellular fibrosis and tissue tension [60]. This intriguing finding opens up the possibility to reduce the aggressiveness of PDAC, elicited by an excess of matricellular fibrosis, by blocking or interfering with STAT3 hyperactivity.

However, other proteins found in stromal cells could contribute to promote tumor invasion by different mechanisms. Caveolin-1 is a scaffolding protein highly expressed in stromal cells involved in a plethora of cellular processes, such as cell proliferation, survival, motility and migration, via the modulation of a variety of signaling pathways aberrantly activated in cancer cells [61,62]. An interesting link has been found between hypoxia in cancer cells and caveolin-1; in HeLa cells this protein regulates the availability of cell surface proteins and endocytosis in hypoxic conditions thus disclosing potential implications for the identification of novel targets in tumor microenvironment [63]. A loss of caveolin-1 in fibroblasts induces a CAF phenotype in the tumor microenvironment and drives the constitutive activation of several oncogenes, such as c-Myc, v-Abl, v-Src, H-Ras, and Neu/ErB2 in stromal fibroblasts. This process results in a myofibroblast conversion with increased deposition of ECM components and hyperactivation of TGF-β signaling pathway, thereby promoting EMT in cancer cells [64].

Another ECM remodeling mechanism in cancer has been recently described: invadopodia are actin-rich membrane protrusions with a marked matrix degradation activity formed by invasive carcinoma cells [65]. Cortactin (CTTN) is also an important player in invadopodia function and in invadopodia-associated ECM degradation, playing an important role in actin assembly, cytoskeletal arrangement and membrane trafficking [66]. It was reported that miR-182, acting as tumor suppressor, inhibited invadopodia formation in NSCLC by targeting cortactin, thus inhibiting ECM degradation and suppressing the migration and invasion of lung cancer cells [67].

Podosomes are conical-shape structures, rich in actin and adhesion or scaffolding proteins, characterized by membrane protrusions and invaginations that could be spotted on a wide variety of cells including cancer cells, macrophages, dendritic cells, vascular smooth cells and endothelial cells [65]. As suggested by their molecular composition, the primary function of podosomes deals with cellular motility and invasion and therefore deciphering the functional consequences of their interaction with the ECM surrounding the microenvironments is a key issue in oncology. Indeed, although the precise role of podosomes and the agents that regulate their formation and turnover is still poorly understood, early findings obtained from macrophage podosomes point toward a mechanism in ECM remodeling and degradation [65].

## 6. Organ-Specific Pre-Metastatic Niches

### 6.1. Lung

Metastatic cancers such as bladder, breast, colon, kidney, melanoma and sarcoma often spread to the lungs, and several ECM components have been recognized to be involved in this process.

**Figure 2:**
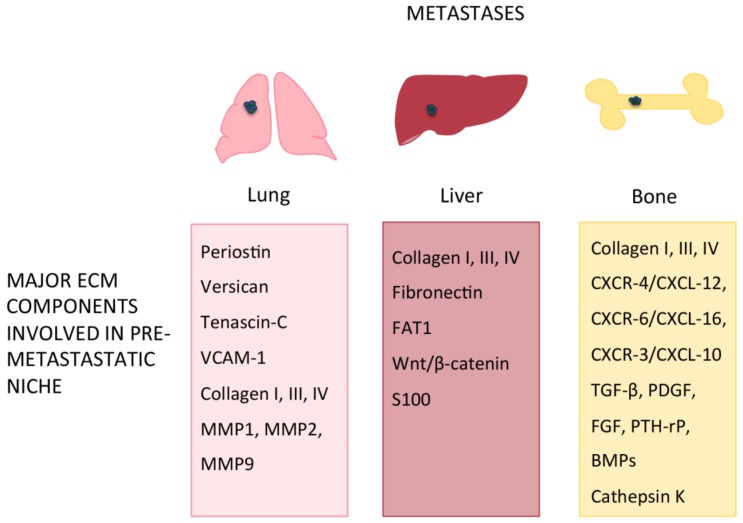
ECM components involved in pre-metastatic niche formation in lung, liver and bone.

Among the ECM proteins involved in the metastatic colonization of lung (Table 2, Figure 2), Tenascin-C (TNC), Periostin (POSTN) and the large chondroitin sulfate proteoglycan Versican (VCAN) have been identified as key players [68,69]. TNC is normally produced by fibroblasts but can also be secreted by breast cancer metastatic cells to create a pre-metastatic niche in the lungs for lung metastasis-initiating processes [70,71] while POSTN, a stromal-derived protein, supports the survival and viability of metastatic cancer stem cells thus allowing these cells to prime the metastatic niche. Similarly, VCAN is secreted by lung cancer cells and by the infiltrating bone marrow-derived myeloid cells CDllbC/Ly6Chigh within metastatic niches in the lung to potentiate lung metastasis [72,73].

Breast cancer cells have developed an interesting mechanism to increase their metastatic potential; these cells produce vascular cell adhesion molecule-1 (VCAM-1), that, upon binding to α4β1 integrin expressed in the pulmonary parenchyma, prepares the soil for the homing of metastatic breast cancer cells [74]. Under certain experimental conditions, melanoma cells may stimulate expression of VCAM-1 directly at pulmonary metastatic sites [75] and VCAM-1 density has been found to modulate melanoma metastatic cell adhesion to the lung [76].

Another mechanism involved in lung pre-metastatic niche formation was reported: toll-like receptors (TLRs), that recognize inflammatory signals and are involved in innate immunity processes [77], have been found to promote metastasis, probably by initiating chronic inflammation. Recently, exosomes-derived small RNAs produced by primary tumors have been found to enhance the secretion of several chemokines and to induce neutrophil recruiting in the PMN, via a molecular mechanism primed by the activation of the TLR3 receptor in lung epithelial cells [77]; this smallRNA-mediated TLR3 activation resulted in a sort of “fertilization” of the lung environment.

In another recent and interesting study, collagen-based ECM production and modifications in lung by breast cancer cells was investigated [78]. In particular, the authors found that pyruvate promotes collagen hydroxylation without affecting its synthesis; the working hypothesis supports the notion that inhibition of pyruvate metabolism could prevent collagen remodeling in the lung ECM thus reducing metastatic growth in the lung.

Several MMPs have been found to take part in lung metastases [79]: MMP-1 overexpression has been found to significantly promote lung metastasis [80]; adenovirus-mediated knockdown of MMP-2 decreased tumor growth and prevented formation of lung nodules [81] while, in a previous study, an increased expression of MMP9 in the premetastatic lung niche derived from a distant primary tumor was found to be induced by VEGFR-1 and to promote lung metastasis [82]. For these reasons, MMPs were identified as possible therapeutic targets to prevent metastasis but, unfortunately, no MMP inhibitor ever resulted in therapeutic application [83].

### 6.2. Liver

All the most important cell types found in the liver, such as stellate cells, sinusoidal endothelial cells and Kupffer cells secrete the main components of ECM, thus underscoring its functional importance in the liver homeostasis and in the pre-metastatic and tumor niche formation [84].

Major alterations of ECM composition related to metastatic niche formation involve a number of proteins with different roles: the cell-adhesion molecule carcinoembryonic antigen (CEA), cell adhesion molecules (CAMs), CXC motif-chemokines (CXCLs), VEGF, MAPK, NF-κB, Citrullinated proteins/PAD, Spermine pullulan, MMPs and collagen isoforms and these alterations may vary dependently from the originating primary tumor [85].

Patients with colorectal liver metastasis (CRLM) have elevated levels of type I collagen in urine and plasma, indicative of an increased collagen turnover in the liver, and specific collagen types derived from primary tumors in the niche (Table 2, Figure 2) [86]. These alterations of collagen turnover, together with changes found in the expression pattern of collagen isoforms, induce changes in ECM composition, thus contributing to prepare and favoring the appropriate soil for tumor seeding [87].

Another study showed a mechanism by which circulating tumor cells (CTCs) adhere to endothelial cells of liver blood vessels using FN deposits as a substrate that allows them to extravasate and form metastasis. In this study, the authors underline that endothelial cells could release FN or accumulate soluble plasma fibronectin produced in situ by hepatocytes. The tumor itself (cancer or stromal cells) could either directly produce FN or stimulate fibronectin production in hepatocytes or liver endothelial cells. In addition, recent evidence has shown that fibronectin upregulation is important for liver pre-metastatic niche formation induced by pancreatic cancer–derived exosomes [88]. Another important mechanism is related to chemotherapeutic agents: in a mouse model, liver metastasis was enhanced by cisplatin or vincristine dependently on cancer cell type [88]. This observation supports the notion that profibrotic microenvironment established by cisplatin or chronic inflammatory condition established by vincristine, may result in a favorable microenvironment capable of promoting metastasis [89]. Another study on collagen IV and liver metastasis showed that upregulation of collagen IV could be a possible driver of metastasis, while downregulating this ECM protein reduces metastasis, indicating tumor-derived ECM type IV collagen as a critical mediator in liver metastasis [90].

In a recent, comprehensive whole-genome study of colorectal metastases, the authors found a higher mutation rate in metastases, compared to matched primary tumors; interestingly, metastases specific mutations were mainly correlated to PI3K-Akt signaling, cell adhesion, ECM and hepatic stellate activation genes, which are critical for homing within the metastatic niche, suggesting the existence of cellular programs for site-specific colonization [91]. Intriguingly, mutations of the FAT atypical cadherin 1 (FAT1), a protein that not only regulates cell invasion but also primes stemness properties, were found to be a unique feature of colorectal metastases. Cadherin-related proteins can interact with β-catenin and segregate it, thereby preventing its nuclear migration and regulating its transcriptional activity. The mechanism by which β-catenin induces cell proliferation and stem cell phenotype is quite complex; β-catenin induces the translocation of T-cell factor (TCF) receptor into the nucleus increasing the transcription of Wnt-target genes [92,93]. The mechanism by which the protocadherin FAT1 acts as a tumor suppressor that relies on its ability to enhance Wnt/β-catenin signaling that could augment metastatic cell malignity [94]. Another report disclosed an additional mechanism by which β-catenin could indirectly promotes epidermal hyperplasia; for example, actomyosin-mediated cellular tension enhances tissue biomechanical properties, such as stiffness, via a β-catenin-mediated activation [56].

### 6.3. Bone

Bone is a complex static structure in which several cell types, such as osteoclasts, osteoblasts, osteocytes and bone marrow stromal cells (BMSC), are embedded and surrounded by a dense ECM network. The bone ECM is composed by an organic compartment, formed by type I collagen, proteoglycans and glycoproteins, and by an inorganic compartment in which calcium and phosphate ions are packed into hydroxyapatite crystals [95]. The bone ECM contributes to different cellular processes necessary for bone life cycle, including cell attachment, differentiation, and migration. Bones could be classified on the basis of their structural features, such as degree of density and porosity, in cortical and trabecular bones; long bones have an interior porous trabecular structure and an external compact coating of cortical bone. Cortical bone is formed by densely packed collagen type I fibrils and is highly mineralized to strengthen the bone structure thus providing a steady support for the skeleton [96], while trabecular bone is a porous matrix organized in a broader structural network [97]. Either in cortical and in trabecular bones, vascularization is provided by sinusoids, large and permeable blood vessels that under specific circumstances allow cell extravasation into the bone tissue [97].

Mesenchymal stem cells give origin to osteoblasts through a differentiation process induced by several soluble factors, such as endothelin-1 (ET-1), platelet-derived growth factor (PDGF), fibroblast growth factor (FGF), bone morphogenetic proteins (BMPs) and transforming growth factor β (TGF-β). Although the main function of osteoblast is osteogenesis, a subpopulation of osteoblasts embedded in the bone matrix, upon acquisition of specific structural functions, can differentiate into osteocytes [98].

Osteoclasts, whose main function deals with bone resorption, derive by differentiation from macrophages; the regulation of osteoclasts activity is quite complex being controlled by a wide variety of extracellular stimuli of different origin. Their activation is induced by 1,25-dihydroxyvitamin D3, parathyroid hormone (PTH) PTH related protein (PTH-rP), interleukin-1 (IL-1), IL-6 and macrophage colony-stimulating factor (MCSF) while other factors, such as calcitonin, IL-4, IL-18 and interferon-β, inhibit their activity [99].

A key role in bone metabolism is played by the receptor activator of nuclear factor-κB (NF-κB) ligand (RANKL): RANKL belongs to the tumor necrosis factor TNFα superfamily and binds to a membrane receptor, named receptor activator of nuclear factor-κB (RANK). RANKL, once produced by osteoblasts and stromal T-cells, stimulates osteoclasts activity, while its interaction with the soluble receptor osteoprotegerin (OPG) turns off RANKL-dependent signaling pathways by blocking RANKL binding to RANK [100].

The bone microenvironment is particularly suitable for bone metastasis (BM) dissemination and a number of different primary tumors tend to metastasize to bone tissue, even at early stages, by stimulating BMSC to prepare pre-metastatic niches. As a result of BM dissemination, bone remodeling is altered with consequences even on the bone matrix and bone structure, such as brittle bones and bone fractures. The disarrangement of collagen/apatite microstructure in BM was found to deeply affect bone mechanical properties; in a recent study the authors found that interactions between cancer cells and osteoblasts disorganize the osteoblast alignment, alter cell-cell and cell-ECM interactions thus leading to a deteriorated bone tissue (Table 2, Figure 2) [101].

Another mechanism by which cancer cells in BM alter the surrounding ECM is the production of enzymes that modify the chemical composition and structure of ECM molecules. The secretion of the enzyme lysyl oxidase (LOX) is upregulated in BM cancer cells, resulting in collagen hydroxilation grade changes that in turn alter tissue stiffness [102], a key factor in cancer growth.

After extravasation in bone, cancer cells become quiescent and express osteoblast or osteoclast cell surface markers (osteomimicry) to escape immune surveillance [103]. These cancer cells nested in bone ECM have been found to overexpress chemokine receptors, such as CXCR-4, whose ligand CXCL-12, or SDF-1, is secreted by BMSC. However, other chemokines, such as CXCR-6/CXCL-16 and CXCR-3/CXCL-10, have also been found to be involved in the process of tumor niche formation [104]. During this stage of apparent dormancy, cancer cells recruit other cells present in the bone microenvironment, such as fibroblasts or osteoblasts, to promote collagen I, III, IV and FN deposition thus leading to ECM disorganization characterized by changes in ECM dynamics and structural properties [97].

Once the surrounding environment appears to be favorable, cancer cells exit the quiescent state and start to proliferate, thus leading to actual BM formation. Based on their different mechanisms and effects on bone ECM, BM can be classified into two main types: lytic and sclerotic. In lytic BM, cytokines promoting osteoclasts activity are secreted by cancer cells to stimulate bone resorption; during this process, growth factors physiologically embedded in the ECM, such as TGFβ, PDGF, FGF, are released and, in turn, stimulate cancer cell proliferation [104,105]. Several studies have demonstrated that a pharmacological approach targeting different molecular mechanisms could be fruitful in the management of BM. In a mouse model of breast cancer BM, a monoclonal antibody neutralizing parathyroid hormone-related protein (PTH-rP), a potent stimulator of osteoclastic bone resorption, suppressed BM [106]; in addition, ibandronate and tisedronate, two bisphosphonate analogues that are specific inhibitors of osteoclastic bone resorption, inhibited osteolytic bone metastases in women with breast cancer receiving hormonal therapy [107,108].

Another molecule that plays a key role during bone resorption in lytic BM onset is cathepsin K. Cathepsin K is a papain-like cysteine protease involved in bone remodeling, highly expressed in osteoclasts and in activated reactive macrophages [109]; this protease affects cancer progression in several tumor types such as breast carcinoma and in prostate cancer [110].

These findings have stimulated efforts in designing and screening new synthetic inhibitors of cathepsin K that are currently under preclinical evaluation [111], even though the recent history of other cathepsin K inhibitors was troubled. Odanacatib, displaying efficacy in inhibiting bone resorption and increasing bone mineral density, also increased the risk of stroke and was dropped during phase III clinical trial. Several other cathepsin inhibitors have previously failed: balicatib was dropped because of dermatological adverse events and relacatib was dropped because of off-target toxicity [112].

Sclerotic BM growth factors secreted by cancer cells, such as TGFβ, BMPs, FGF and Wnt, induce osteoblast differentiation and activity, while ET-1 inhibits osteoclasts activity [97], thus shifting the bone metabolism towards a blastic uncontrolled behavior. Prostate cancer commonly gives rise to BM and prostate cancer cells induce the transition from endothelial cells to osteoblasts by releasing BMP-4 in the bone marrow [98,113]; however, this issue is far from being clarified because in most patients both lytic and sclerotic BM are present, suggesting that a real boundary between the two mechanisms cannot be actually identified [114].

Recently, the role of several factors leading to BM by a “vicious cycle” has been characterized; PTHrP secreted by tumor cells stimulates osteoblasts and prime osteolysis; concomitantly, upregulation of the RANKL-dependent signaling pathway in osteoblasts leads to osteoclasts activation and bone resorption [115]. The activated osteoclasts subsequently degrade the bone matrix releasing proteinases, such as cathepsin K, MMP-9, and MMP-13 and growth factors such as TGFβ, IGF1. These growth factors, together with the release of hydrogen ions to create a strong acid microenvironment, stimulate tumor growth and induce the release of tumor derived PTHrP [116], thereby reinforcing “the vicious cycle”.

## 7. Conclusions

The impact of metastasis on cancer mortality is still dramatic, and an extensive effort by the scientific community is mandatory in this field. Recently, new insights have emerged in the identification of circulating metastatic cells because of great improvement in liquid biopsies techniques and applications. However, it appears that the most easily targetable steps in metastasis deal with the formation of premetastatic niche and colonization of distant sites—two mechanisms that deeply imply the involvement of ECM.

Unfortunately, previous pharmacological approaches based on single molecules aimed at inhibiting cell attachment by targeting interaction of surface receptors with ECM components or by disturbing specific signaling pathways gave disappointing results or limited benefits.

On the basis of recent studies, three different approaches may be hypothesized to counteract metastatic processes.

The first option exploits the possibility, already working in other pathologies, that treatment with multiple agents, targeting different mechanisms in an aberrant signaling pathway, could be much more effective in preventing or shrinking the metastatic process compared to single molecule administration. Moreover, therapeutic schemes taking into account early timing and accumulating dose of combined drugs could be screened in appropriate in vitro settings to ascertain whether these combined treatments affect the composition and subsequent functionality of ECM.

Another approach could be focused on the potential “educational” role of EV secreted by metastatic cells in directing and changing the properties of the “soil” in distant sites to colonize. Blocking or interfering with EV uptake might be a fruitful strategy to understand the role of these vesicles in instructing the target cells to modify their pattern of expression of ECM. The multidrug approach, together with the EV-discovery approach, could be now tested and screened using novel outstanding in vitro models such 3D cell (co)cultures, cancer organoids and cancer-on-a-chip technologies. The main advantages of these models rely on the possibility of mimicking very closely the interplay among all the cancer and non-cancer cells belonging to the metastatic niche and observing the consequences of induced ECM modifications on the functionality of these cells.

Finally, a giant leap forward in our knowledge on the molecular events that govern the interplay between ECM and cancer cells could come from innovative single-cell multiomics technologies. Indeed, comparing healthy vs. cancerous human specimens obtained from surgery by spatial genomic or transcriptomic could hold great promise in characterizing the dynamic changes occurring at different stages in the formation of premetastatic niche.

These considerations, stressing the complexity and the difficulty of the fight against metastasis, indicate that only a synergistic huge collaboration among the pharmaceutical industry, academic and government organizations will have better chances in the near future to defeat metastasis. 

## Figures and Tables

**Table 1:** Major ECM components involved in pre-metastatic niche formation in lung, liver and bone that are proteinase substrates.

ECM Component	Proteinase
Versican (VCAN)	ADAMTs
Collagen I	MMP1, MMP3, MMP8, MMP10, MMP11, MMP13, MMP14, MMP19 ADAM8
Collagen III	MMP1, MMP3, MMP8, MMP9, MMP10, MMP11, MMP13, MMP14, MMP16,
Collagen IV	MMP1, MMP2, MMP3, MMP7, MMP8, MMP9, MMP10, MMP11, MMP12, MMP13, MMP14, MMP19, MMP25, MMP26, ADAM15 Meprins
Fibronectin	MMP2, MMP7, MMP8, MMP9, MMP10, MMP11, MMP12, MMP13, MMP14, MMP15, MMP16, MMP19, MMP24, MMP25, MMP26 ADAM8, ADAM9, ADAM12, ADAMTs Meprins
TGF-β	Plasmin, MMP2, MMP9
Periostin	ADAM8

MMP, matrix metalloproteinase; TGF-β, transforming growth factor β; ADAMs, a disintegrin and metalloproteinases; ADAMTs, ADAMs with a thrombospondin motif (for extensive review see [1]).

**Table 2:** Major ECM components or mediators involved in pre-metastatic niche (PMN) formation in lung, liver and bone.

ECM Component	PMN Site	Mechanism	Reference
Periostin (POSTN)	Lung	Induces EMT, promotes lung metastases	[54]
Versican (VCAN)	Lung	Secreted by the infiltrating bone marrow-derived myeloid cells, promotes metastatic niche formation	[55]
Tenascin-C (TNC)	Lung	Secreted by breast cancer cells, promotes PMN formation	[56,57]
Vascular cell adhesion molecule-1 (VCAM-1)	Lung	Binds to α4β1 integrin, promotes homing of breast and melanoma cancer cells	[60,61,62]
Collagen I, III, IV	Lung, liver, bone	Increased collagen hydroxylation promotes ECM remodeling; increased collagen I turnover in hepatic metastases	[64,72,73,84,88]
MMP-1, MMP-2, MMP-9	Lung	Increased MMPs expression promotes PMN formation	[65,66,67,68]
Fibronectin	Liver	Increased production in liver PMN; facilitates CTC extravasation	[74,75,76]
Atypical Cadherin 1 (FAT1)	Liver	Inactivation in liver metastases	[77]
Wnt/β-catenin axis	Liver	Aberrant signaling in liver metastases	[79,80,81]
CXCR-4/CXCL-12, CXCR-6/CXCL-16, CXCR-3/CXCL-10,	Bone	Promote bone metastases	[90]
TGF-β, PDGF, FGF, PTH-rP, BMPs Cathepsin K	Bone	Released during ECM degradation, promote bone metastases	[91,92,93,94]

CTC, circulating tumor cells; MMP, matrix metalloproteinase; TGF-β, transforming growth factor β; PDGF, platelet derived growth factor; FGF, fibroblast growth factor; BMPs, bone morphogenetic proteins; PTH-rP, parathyroid hormone (PTH) related protein.

## References

[1] Bonnans C., Chou J., Werb Z. (2014). Remodelling the extracellular matrix in development and disease. Nat. Rev. Mol. Cell Biol..

[2] Lu P., Weaver V.M., Werb Z. (2012). The extracellular matrix: A dynamic niche in cancer progression. J. Cell Biol..

[3] Schaefer L., Schaefer R.M. (2010). Proteoglycans: From structural compounds to signaling molecules. Cell Tissue Res..

[4] Järveläinen H., Sainio A., Koulu M., Wight T.N., Penttinen R. (2009). Extracellular matrix molecules: Potential targets in pharmacotherapy. Pharmacol. Rev..

[5] Gordon M.K., Hahn R.A. (2010). Collagens. Cell Tissue Res..

[6] Vindin H., Mithieux S.M., Weiss A.S. (2019). Elastin architecture. Matrix Biol..

[7] Oxford J.T., Reeck J.C., Hardy M.J. (2019). Extracellular Matrix in Development and Disease. Int. J. Mol. Sci..

[8] Paolillo M., Schinelli S. (2017). Integrins and exosomes, a dangerous liaison in cancer progression. Cancers.

[9] Paolillo M., Galiazzo M.C., Daga A., Ciusani E., Serra M., Colombo L., Schinelli S. (2018). An RGD small-molecule integrin antagonist induces detachment-mediated anoikis in glioma cancer stem cells. Int. J. Oncol..

[10] Fouad Y.A., Aanei C. (2017). Revisiting the hallmarks of cancer. Am. J. Cancer Res..

[11] Foster D.S., Jones R.E., Ransom R.C., Longaker M.T., Norton J.A. (2018). The evolving relationship of wound healing and tumor stroma. JCI Insight.

[12] Schäfer M., Werner S. (2008). Cancer as an overhealing wound: An old hypothesis revisited. Nat. Rev. Mol. Cell Biol..

[13] Micalizzi D.S., Maheswaran S., Haber D.A. (2017). A conduit to metastasis: Circulating tumor cell biology. Genes Dev..

[14] Pradella D., Naro C., Sette C., Ghigna C. (2017). EMT and stemness: Flexible processes tuned by alternative splicing in development and cancer progression. Mol. Cancer.

[15] Kang K.W., Lee M.J., Song J.A., Jeong J.Y., Kim Y.K., Lee C., Kim T.H., Kwak K.B., Kim O.J., An H.J. (2014). Overexpression of goosecoid homeobox is associated with chemoresistance and poor prognosis in ovarian carcinoma. Oncol. Rep..

[16] Jolly M.K., Ware K.E., Gilja S., Somarelli J.A., Levine H. (2017). EMT and MET: Necessary or permissive for metastasis?. Mol. Oncol..

[17] Chin A.R., Wang S.E. (2016). Cancer tills the Premetastatic field: Mechanistic basis and clinical implications. Clin. Cancer Res..

[18] Liu Y., Cao X. (2016). Characteristics and Significance of the Pre-metastatic Niche. Cancer Cell.

[19] Hiratsuka S., Watanabe A., Aburatani H., Maru Y. (2006). Tumour mediated upregulation of chemoattractants and recruitment of myeloid cells predetermines lung metastasis. Nat. Cell Biol..

[20] Hiratsuka S., Watanabe A., Sakurai Y., Akashi-Takamura S., Ishibashi S., Miyake K., Shibuya M., Akira S., Aburatani H., Maru Y. (2008). The S100A8-serum amyloid A3-TLR 4 paracrine cascade establishes a pre-metastatic phase. Nat. Cell Biol..

[21] Li Y., Kong F., Jin C., Hu E., Shao Q., Liu J., He D., Xiao X. (2019). The expression of S100A8/S100A9 is inducible and regulated by the Hippo/YAP pathway in squamous cell carcinomas. BMC Cancer.

[22] Wang Y., Ding Y., Guo N., Wang S. (2019). MDSCs: Key Criminals of Tumor Pre-metastatic Niche Formation. Front. Immunol..

[23] Wang J.P., Hielscher A. (2017). Fibronectin: How Its Aberrant Expression in Tumors May Improve Therapeutic Targeting. J. Cancer.

[24] Hoshino A., Costa-Silva B., Shen T.L., Rodrigues G., Hashimoto A., Tesic Mark M., Molina H., Kohsaka S., Di Giannatale A., Ceder S. (2015). Tumour exosome integrins determine organotropic metastasis. Nature.

[25] Høye A.M., Erler J.T. (2016). Structural ECM components in the premetastatic and metastatic niche. Am. J. Physiol. Cell Physiol..

[26] Liu T., Zhou L., Li D., Andl T., Zhang Y. (2019). Cancer-Associated Fibroblasts Build and Secure the Tumor Microenvironment. Front. Cell Dev. Biol..

[27] Wei S.C., Fattet L., Tsai J.H., Guo Y., Pai V.H., Majeski H.E., Chen A.C., Sah R.L., Taylor S.S., Engler A.J. (2015). Matrix stiffness drives epithelial-mesenchymal transition and tumour metastasis through a twist1-g3bp2 mechanotransduction pathway. Nat. Cell Biol..

[28] Walker C., Mojares E., Del Río Hernández A. (2018). Role of Extracellular Matrix in Development and Cancer Progression. Int. J. Mol. Sci..

[29] Sekiya S., Miura S., Matsuda-Ito K., Suzuki A. (2016). Myofibroblasts Derived from Hepatic Progenitor Cells Create the Tumor Microenvironment. Stem Cell Rep..

[30] Saini F., Argent R.H., Grabowska A.M. (2019). Sonic Hedgehog Ligand: A Role in Formation of a Mesenchymal Niche in Human Pancreatic Ductal Adenocarcinoma. Cells.

[31] Hanley C.J., Noble F., Ward M., Bullock M., Drifka C., Mellone M., Manousopoulou A., Johnston H.E., Hayden A., Thirdborough S. (2016). A subset of myofibroblastic cancer-associated fibroblasts regulate collagen fiber elongation, which is prognostic in multiple cancers. Oncotarget.

[32] Alkasalias T., Moyano-Galceran L., Arsenian-Henriksson M., Lehti K. (2018). Fibroblasts in the tumor microenvironment: Shield or spear?. Int. J. Mol. Sci..

[33] Chanmee T., Ontong P., Konno K., Itano N. (2014). Tumor-associated macrophages as major players in the tumor microenvironment. Cancers.

[34] Poh A.R., Ernst M. (2018). Targeting Macrophages in Cancer: From Bench to Bedside. Front. Oncol..

[35] Afik R., Zigmond E., Vugman M., Klepfish M., Shimshoni E., Pasmanik-Chor M., Shenoy A., Bassat E., Halpern Z., Geiger T. (2016). Tumor macrophages are pivotal constructors of tumor collagenous matrix. J. Exp. Med..

[36] Chantrain C.F., Henriet P., Jodele S., Emonard H., Feron O., Courtoy P.J., DeClerck Y.A., Marbaix E. (2006). Mechanisms of pericyte recruitment in tumour angiogenesis: A new role for metalloproteinases. Eur. J. Cancer.

[37] Armulik A., Genove G., Betsholtz C. (2011). Pericytes: Developmental, physiological, and pathological perspectives, problems, and promises. Dev. Cell.

[38] De Palma M., Biziato D., Petrova T.V. (2017). Microenvironmental regulation of tumour angiogenesis. Nat. Rev. Cancer.

[39] Thijssen V.L., Paulis Y.W., Nowak-Sliwinska P., Deumelandt K.L., Hosaka K., Soetekouw P.M., Cimpean A.M., Raica M., Pauwels P., van den Oord J.J. (2018). Targeting PDGF-mediated recruitment of pericytes blocks vascular mimicry and tumor growth. J. Pathol..

[40] You W.K., Stallcup W.B. (2017). Localization of VEGF to Vascular ECM Is an Important Aspect of Tumor Angiogenesis. Cancers.

[41] Arandkar S., Furth N., Elisha Y., Nataraj N.B., van der Kuip H., Yarden Y., Aulitzky W., Ulitsky I., Geiger B., Oren M. (2018). Altered p53 functionality in cancer-associated fibroblasts contributes to their cancer-supporting features. Proc. Natl. Acad. Sci. USA.

[42] Najafi M., Farhood B., Mortezaee K. (2019). Extracellular matrix (ECM) stiffness and degradation as cancer drivers. J. Cell Biochem..

[43] Henriet P., Zhong Z.D., Brooks P.C., Weinberg K.I., DeClerck Y.A. (2000). Contact with fibrillar collagen inhibits melanoma cell proliferation by up-regulating p27 KIP1. Proc. Natl. Acad. Sci. USA.

[44] Gonzalez-Avila G., Sommer B., Mendoza-Posada D.A., Ramos C., Garcia-Hernandez A.A., Falfan-Valencia R. (2019). Matrix metalloproteinases participation in the metastatic process and their diagnostic and therapeutic applications in cancer. Crit. Rev. Oncol. Hematol..

[45] Apte R.S., Chen D.S., Ferrara N. (2019). VEGF in Signaling and Disease: Beyond Discovery and Development. Cell.

[46] Conrad C., Benzel J., Dorzweiler K., Cook L., Schlomann U., Zarbock A., Slater E.P., Nimsky C., Bartsch J.W. (2019). ADAM8 in invasive cancers: Links to tumor progression, metastasis, and chemoresistance. Clin. Sci. (Lond.).

[47] Romagnoli M., Mineva N.D., Polmear M., Conrad C., Srinivasan S., Loussouarn D., Barillé-Nion S., Georgakoudi I., Dagg Á., McDermott E.W. (2014). ADAM8 expression in invasive breast cancer promotes tumor dissemination and metastasis. EMBO Mol. Med..

[48] Conrad C., Götte M., Schlomann U., Roessler M., Pagenstecher A., Anderson P., Preston J., Pruessmeyer J., Ludwig A., Li R. (2018). ADAM8 expression in breast cancer derived brain metastases: Functional implications on MMP-9 expression and transendothelial migration in breast cancer cells. Int. J. Cancer.

[49] Li W., Wang D., Sun X., Zhang Y., Wang L., Suo J. (2019). ADAM17 promotes lymph node metastasis in gastric cancer via activation of the Notch and Wnt signaling pathways. Int. J. Mol. Med..

[50] Geurts N., Becker-Pauly C., Martens E., Proost P., Van den Steen P.E., Stöcker W., Opdenakker G. (2012). Meprins process matrix metalloproteinase-9 (MMP-9)/gelatinase B and enhance the activation kinetics by MMP-3. FEBS Lett..

[51] Broder C., Becker-Pauly C. (2013). The metalloproteases meprin α and meprin β: Unique enzymes in inflammation, neurodegeneration, cancer and fibrosis. Biochem. J..

[52] Vadon-Le Goff S., Hulmes D.J., Moali C. (2015). BMP-1/tolloid-like proteinases synchronize matrix assembly with growth factor activation to promote morphogenesis and tissue remodeling. Matrix Biol..

[53] Zhang K., Corsa C.A., Ponik S.M., Prior J.L., Piwnica-Worms D., Eliceiri K.W., Keely P.J., Longmore G.D. (2013). The collagen receptor discoidin domain receptor 2 stabilizes SNAIL1 to facilitate breast cancer metastasis. Nat. Cell Biol..

[54] Xiao W., Wang X., Wang T., Xing J. (2019). Overexpression of BMP1 reflects poor prognosis in clear cell renal cell carcinoma. Cancer Gene Ther..

[55] Winer A., Adams S., Mignatti P. (2018). Matrix Metalloproteinase Inhibitors in cancer therapy: Turning past failures into future successes. Mol. Cancer Ther..

[56] Samuel M.S., Lopez J.I., McGhee E.J., Croft D.R., Strachan D., Timpson P., Munro J., Schröder E., Zhou J., Brunton V.G. (2011). Actomyosin-mediated cellular tension drives increased tissue stiffness and β-catenin activation to induce epidermal hyperplasia and tumor growth. Cancer Cell.

[57] Levental K.R., Yu H., Kass L., Lakins J.N., Egeblad M., Erler J.T., Fong S.F., Csiszar K., Giaccia A., Weninger W. (2009). Matrix crosslinking forces tumor progression by enhancing integrin signaling. Cell.

[58] Mouw J.K., Yui Y., Damiano L., Bainer R.O., Lakins J.N., Acerbi I., Ou G., Wijekoon A.C., Levental K.R., Gilbert P.M. (2014). Tissue mechanics modulate microRNA-dependent PTEN expression to regulate malignant progression. Nat. Med..

[59] Tan F.H., Bai Y., Saintigny P., Darido C. (2019). mTOR Signalling in Head and Neck Cancer: Heads Up. Cells.

[60] Laklai H., Miroshnikova Y.A., Pickup M.W., Collisson E.A., Kim G.E., Barrett A.S., Hill R.C., Lakins J.N., Schlaepfer D.D., Mouw J.K. (2016). Genotype tunes pancreatic ductal adenocarcinoma tissue tension to induce matricellular fibrosis and tumor progression. Nat. Med..

[61] Díaz-Valdivia N.I., Calderón C.C., Díaz J.E., Lobos-González L., Sepulveda H., Ortíz R.J., Martinez S., Silva V., Maldonado H.J., Silva P. (2017). Anti-neoplastic drugs increase caveolin-1-dependent migration, invasion and metastasis of cancer cells. Oncotarget.

[62] Ketteler J., Klein D. (2018). Caveolin-1, cancer and therapy resistance. Int. J. Cancer.

[63] Bourseau-Guilmain E., Menard J.A., Lindqvist E., Indira Chandran V., Christianson H.C., Cerezo Magaña M., Lidfeldt J., Marko-Varga G., Welinder C., Belting M. (2016). Hypoxia regulates global membrane protein endocytosis through caveolin-1 in cancer cells. Nat. Commun..

[64] Shen X.J., Zhang H., Tang G.S., Wang X.D., Zheng R., Wang Y., Zhu Y., Xue X.C., Bi J.W. (2015). Caveolin-1 is a modulator of fibroblast activation and a potential biomarker for gastric cancer. Int. J. Biol. Sci..

[65] Yamaguchi H., Pixley F., Condeelis J. (2006). Invadopodia and podosomes in tumor invasion. Eur. J. Cell Biol..

[66] Clark E.S., Weaver A.M. (2008). A new role for cortactin in invadopodia: Regulation of protease secretion. Eur. J. Cell Biol..

[67] Li Y., Zhang H., Gong H., Yuan Y., Li Y., Wang C., Li W., Zhang Z., Liu M., Liu H. (2018). miR-182 suppresses invadopodia formation and metastasis in non-small cell lung cancer by targeting cortactin gene. J. Exp. Clin. Cancer Res..

[68] Soikkeli J., Podlasz P., Yin M., Nummela P., Jahkola T., Virolainen S., Krogerus L., Heikkilä P., von Smitten K., Saksela O. (2010). Metastaticoutgrowth encompasses COL-I, FN1, and POSTN up-regulation and assembly to fibrillar networks regulating cell adhesion, migration, and growth. Am. J. Pathol..

[69] Asano K., Nelson C.M., Nandadasa S., Aramaki-Hattori N., Lindner D.J., Alban T., Inagaki J., Ohtsuki T., Oohashi T., Apte S.S. (2017). Stromal Versican Regulates Tumor Growth by Promoting Angiogenesis. Sci. Rep..

[70] Oskarsson T., Acharyya S., Zhang X.H., Vanharanta S., Tavazoie S.F., Morris P.G., Downey R.J., Manova-Todorova K., Brogi E., Massague J. (2011). Breast cancer cells produce tenascin C as a metastatic niche component to colonize the lungs. Nat. Med..

[71] O’Connell J.T., Sugimoto H., Cooke V.G., MacDonald B.A., Mehta A.I., LeBleu V.S., Dewar R., Rocha R.M., Brentani R.R., Resnick M.B. (2011). VEGF-A and Tenascin-C produced by S100A4C stromal cells are important for metastatic colonization. Proc. Natl. Acad. Sci. USA.

[72] Malanchi I., Santamaria-Martine A., Susanto E., Peng H., Lehr H.A., Delaloye J.F., Huelsken J. (2012). Interactions between cancer stem cells and their niche govern metastatic colonization. Nature.

[73] Gao D., Joshi N., Choi H., Ryu S., Hahn M., Catena R., Sadik H., Argani P., Wagner P., Vahdat L.T. (2012). Myeloid progenitor cells in the premetastatic lung promote metastases by inducing mesenchymal to epithelial transition. Cancer Res..

[74] Minn A.J., Gupta G.P., Siegel P.M., Bos P.D., Shu W., Giri D.D., Viale A., Olshen A.B., Gerald W.L., Massague J. (2005). Genes that mediate breast cancer metastasis to lung. Nature.

[75] Langley R.R., Carlisle R., Ma L., Specian R.D., Gerritsen M.E., Granger D.N. (2001). Endothelial expression of vascular cell adhesion molecule-1 correlates with metastatic pattern in spontaneous melanoma. Microcirculation.

[76] Amschler K., Kossmann E., Erpenbeck L., Kruss S., Schill T., Schön M., Möckel S.M.C., Spatz J.P., Schön M.P. (2018). Nanoscale Tuning of VCAM-1 Determines VLA-4-Dependent Melanoma Cell Plasticity on RGD Motifs. Mol. Cancer Res..

[77] Liu Y., Gu Y., Han Y., Zhang Q., Jiang Z., Zhang X., Huang B., Xu X., Zheng J., Cao X. (2016). Tumor Exosomal RNAs Promote Lung Pre-metastatic Niche Formation by Activating Alveolar Epithelial TLR3 to Recruit Neutrophils. Cancer Cell.

[78] Elia I., Rossi M., Stegen S., Broekaert D., Doglioni G., van Gorsel M., Boon R., Escalona-Noguero C., Torrekens S., Verfaillie C. (2019). Breast cancer cells rely on environmental pyruvate to shape the metastatic niche. Nature.

[79] Merchant N., Nagaraju G.P., Rajitha B., Lammata S., Jella K.K., Buchwald Z.S., Lakka S.S., Ali A.N. (2017). Matrix metalloproteinases: Their functional role in lung cancer. Carcinogenesis.

[80] Fanjul-Fernández M., Folgueras A.R., Fueyo A., Balbín M., Suárez M.F., Fernández-García M.S., Shapiro S.D., Freije J.M.P., López-Otín C. (2018). Matrix metalloproteinase Mmp-1a is dispensable for normal growth and fertility in mice and promotes lung cancer progression by modulating inflammatory responses. J. Biol. Chem..

[81] Tsung A.J., Kargiotis O., Chetty C., Lakka S.S., Gujrati M., Spomar D.G., Dinh D.H., Rao J.S. (2008). Downregulation of matrix metalloproteinase-2 (MMP-2) utilizing adenovirus-mediated transfer of small interfering RNA (siRNA) in a novel spinal metastatic melanoma model. Int. J. Oncol..

[82] Hiratsuka S., Nakamura K., Iwai S., Murakami M., Itoh T., Kijima H., Shipley J.M., Senior R.M., Shibuya M. (2002). MMP9 induction by vascular endothelial growth factor receptor-1 is involved in lung-specific metastasis. Cancer Cell.

[83] Zhong Y., Lu Y.T., Sun Y., Shi Z.H., Li N.G., Tang Y.P., Duan J.A. (2018). Recent opportunities in matrix metalloproteinase inhibitor drug design for cancer. Expert Opin. Drug Discov..

[84] Williamson T., Sultanpuram N., Sendi H. (2019). The role of liver microenvironment in hepatic metastasis. Clin. Transl. Med..

[85] Azizidoost S., Ahmadzadeh A., Rahim F., Shahjahani M., Seghatoleslami M., Saki N. (2016). Hepatic metastatic niche: From normal to pre-metastatic and metastatic niche. Tumour Biol..

[86] Kahlert C., Pecqueux M., Halama N., Dienemann H., Muley T., Pfannschmidt J., Lasitschka F., Klupp F., Schmidt T., Rahbari N. (2014). Tumour-site-dependent expression profile of angiogenic factors in tumourassociated stroma of primary colorectal cancer and metastases. Br. J. Cancer.

[87] van Huizen N.A., Coebergh van den Braak R.R.J., Doukas M., Dekker L.J.M., IJzermans J.N.M., Luider T.M. (2019). Up-regulation of collagen proteins in colorectal liver metastasis compared with normal liver tissue. J. Biol. Chem..

[88] Barbazán J., Alonso-Alconada L., Elkhatib N., Geraldo S., Gurchenkov V., Glentis A., van Niel G., Palmulli R., Fernández B., Viaño P. (2017). Liver Metastasis Is Facilitated by the Adherence of Circulating Tumor Cells to Vascular Fibronectin Deposits. Cancer Res..

[89] Zenitani M., Nojiri T., Hosoda H., Kimura T., Uehara S., Miyazato M., Okuyama H., Kangawa K. (2018). Chemotherapy can promote liver metastasis by enhancing metastatic niche formation in mice. J. Surg. Res..

[90] Burnier J.V., Wang N., Michel R.P., Hassanain M., Li S., Lu Y., Metrakos P., Antecka E., Burnier M.N., Ponton A. (2011). Type IV collagen-initiated signals provide survival and growth cues required for liver metastasis. Oncogene.

[91] Ishaque N., Abba M.L., Hauser C., Patil N., Paramasivam N., Huebschmann D., Leupold J.H., Balasubramanian G.P., Kleinheinz K., Toprak U.H. (2018). Whole genome sequencing puts forward hypotheses on metastasis evolution and therapy in colorectal cancer. Nat. Commun..

[92] Lustig B., Behrens J. (2003). The Wnt signaling pathway and its role in tumor development. J. Cancer Res. Clin. Oncol..

[93] Zhan T., Ambrosi G., Wandmacher A.M., Rauscher B., Betge J., Rindtorff N., Häussler R.S., Hinsenkamp I., Bamberg L., Hessling B. (2019). MEK inhibitors activate Wnt signalling and induce stem cell plasticity in colorectal cancer. Nat. Commun..

[94] Morris L.G., Kaufman A.M., Gong Y., Ramaswami D., Walsh L.A., Turcan Ş., Eng S., Kannan K., Zou Y., Peng L. (2013). Recurrent somatic mutation of FAT1 in multiple human cancers leads to aberrant Wnt activation. Nat. Genet..

[95] Florencio-Silva R., Sasso G.R., Sasso-Cerri E., Simões M.J., Cerri P.S. (2015). Biology of Bone Tissue: Structure, Function, and Factors That Influence Bone Cells. Biomed. Res. Int..

[96] Bayraktar H.H., Morgan E.F., Niebur G.L., Morris G.E., Wong E.K., Keaveny T.M. (2004). Comparison of the elastic and yield properties of human femoral trabecular and cortical bone tissue. J. Biomech..

[97] Kolb A.D., Bussard K.M. (2019). The Bone Extracellular Matrix as an Ideal Milieu for Cancer Cell Metastases. Cancers.

[98] D’Oronzo S., Coleman R., Brown J., Silvestris F. (2018). Metastatic bone disease: Pathogenesis and therapeutic options: Up-date on bone metastasis management. J. Bone Oncol..

[99] D’Oronzo S., Brown J., Coleman R. (2017). The role of biomarkers in the management of bone-homing malignancies. J. Bone Oncol..

[100] Rao S., Cronin S.J.F., Sigl V., Penninger J.M. (2018). RANKL and RANK: From Mammalian Physiology to Cancer Treatment. Trends Cell Biol..

[101] Matsugaki A., Harada T., Kimura Y., Sekita A., Nakano T. (2018). Dynamic collision behavior between osteoblasts and tumor cells regulates the disordered arrangement of collagen fiber/apatite crystals in metastasized bone. Int. J. Mol. Sci..

[102] Wang T.H., Hsia S.M., Shieh T.M. (2016). Lysyl Oxidase and the Tumor Microenvironment. Int. J. Mol. Sci..

[103] Kan C., Vargas G., Pape F.L., Clézardin P. (2016). Cancer Cell colonisation in the bone microenvironment. Int. J. Mol. Sci..

[104] Coniglio S.J. (2018). Role of Tumor-Derived Chemokines in Osteolytic Bone Metastasis. Front. Endocrinol. (Lausanne).

[105] Esposito M., Guise T., Kang Y. (2018). The Biology of Bone Metastasis. Cold Spring Harb. Perspect. Med..

[106] Guise T.A., Yin J.J., Taylor S.D., Kumagai Y., Dallas M., Boyce B.F., Yoneda T., Mundy G.R. (1996). Evidence for a causal role of parathyroid hormone-related protein in the pathogenesis of human breast cancer-mediated osteolysis. J. Clin. Investig..

[107] Lester J.E., Dodwell D., Purohit O.P., Gutcher S.A., Ellis S.P., Thorpe R., Horsman J.M., Coleman R.E. (2008). Prevention of anastrozole-induced bone loss with monthly oral ibandronate during adjuvant aromatase inhibitor therapy for breast cancer. Clin. Cancer Res..

[108] von Moos R., Costa L., Gonzalez-Suarez E., Terpos E., Niepel D., Body J.J. (2019). Management of bone health in solid tumours: From bisphosphonates to a monoclonal antibody. Cancer Treat. Rev..

[109] Yasuda Y., Li Z., Greenbaum D., Bogyo M., Weber E., Brömme D. (2004). Cathepsin V, a novel and potent elastolytic activity expressed in activated macrophages. J. Biol. Chem..

[110] Munari E., Cima L., Massari F., Bertoldo F., Porcaro A.B., Caliò A., Riva G., Ciocchetta E., Ciccarese C., Modena A. (2017). Cathepsin K expression in castration-resistant prostate carcinoma: A therapeutical target for patients at risk for bone metastases. Int. J. Biol. Markers.

[111] Jensen A.B., Wynne C., Ramirez G., He W., Song Y., Berd Y., Wang H., Mehta A., Lombardi A. (2010). The cathepsin K inhibitor odanacatib suppresses bone resorption in women with breast cancer and established bone metastases: Results of a 4-week, double-blind, randomized, controlled trial. Clin. Breast Cancer.

[112] Mullard A. (2016). Merck &Co. drops osteoporosis drug odanacatib. Nat. Rev. Drug Discov..

[113] Lin S.C., Lee Y.C., Yu G., Cheng C.J., Zhou X., Chu K., Murshed M., Le N.T., Baseler L., Abe J.I. (2017). Endothelial-to-osteoblast conversion generates osteoblastic metastasis of prostate cancer. Dev. Cell.

[114] Macedo F., Ladeira K., Pinho F., Saraiva N., Bonito N., Pinto L., Goncalves F. (2017). Bone metastases: An overview. Oncol. Rev..

[115] Chen W., Hoffmann A.D., Liu H., Liu X. (2018). Organotropism: New insights into molecular mechanisms of breast cancer metastasis. NPJ Precis. Oncol..

[116] Lynch C.C. (2011). Matrix metalloproteinases as master regulators of the vicious cycle of bone metastasis. Bone.

